# Use of herbal medicinal products among patients in primary health care in a Brazilian southeastern city: evidence from the Prover project

**DOI:** 10.31744/einstein_journal/2024AO0827

**Published:** 2024-10-21

**Authors:** Betania Barros Cota, Jéssica de Castro Alves, Alberto Araújo de Caux, Leila Cristina Ferreira Passagli, Ana Karine Sarvel de Castro, Tatiana Chama Borges Luz

**Affiliations:** 1 Fundação Oswaldo Cruz Instituto René Rachou Grupo de Estudos Transdisciplinares em Tecnologias em Saúde e Ambiente Belo Horizonte MG Brazil Grupo de Estudos Transdisciplinares em Tecnologias em Saúde e Ambiente, Instituto René Rachou, Fundação Oswaldo Cruz, Belo Horizonte, MG, Brazil.; 2 Universidade Estadual de Campinas Departamento de Química de Produtos Naturais Campinas SP Brazil Departamento de Química de Produtos Naturais, Universidade Estadual de Campinas, Campinas, SP, Brazil.; 3 Pronto Atendimento da Prefeitura de Belo Horizonte Belo Horizonte MG Brazil Pronto Atendimento da Prefeitura de Belo Horizonte, Belo Horizonte, MG, Brazil.; 4 Fundação Oswaldo Cruz Instituto René Rachou Belo Horizonte MG Brazil Instituto René Rachou, Fundação Oswaldo Cruz, Belo Horizonte, MG, Brazil.

**Keywords:** Phytotherapy, Plants, medicinal, Primary health care, Epidemiology, Risk factors, Brazil

## Abstract

**Objective:**

To determine the prevalence and factors associated with herbal medicinal product use among primary healthcare patients in southeastern Brazil.

**Methods:**

A cross-sectional study combined with documentary research was conducted between August and November 2017, involving interviews with 1,221 adults following dispensing from primary healthcare units. The characterization of herbal medicinal product usage included assessing the prevalence, redundancy, and interaction indicators, as well as identifying sources of recommendation and acquisition. Descriptive statistics were used for variable analysis and logistic regression was used to estimate the factors associated with herbal medicinal product usage, calculating odds ratios at a significance level of 5%.

**Results:**

The prevalence of herbal medicinal product usage was 3.8%, with 60% of them prescribed by physicians. Private pharmacies (53%) were the main sources, and *Valeriana officinalis* (42%) was the most cited plant species and the sole herbal medicinal product on the municipal list. The indicators of redundancy and interactions between concurrently prescribed pharmaceutical and herbal medicinal products were 42.9% and 28.6%, respectively. Herbal medicinal products usage correlated with the female sex (OR= 2.50; 95%CI= 1.11-5.59), non-white race (OR= 0.52; 95%CI= 0.28-0.94), monthly income above 1 minimum wage (OR= 3.48; 95%CI= 1.51-8.01), and self-reported of anxiety, mood disorder or depression (OR= 2.97; 95%CI= 1.55-5.66).

**Conclusion:**

Despite the low use of herbal medicinal products, users face a high potential for interactions and therapeutic redundancy. Special attention should be paid to patients taking herbal medicinal products and allopathic medicines concurrently. Women with anxiety, mood disorders, or depression were more likely to use herbal medicinal products, whereas non-white people were 48% less likely to use them, suggesting potential socioeconomic disparities in access.

## In Brief

Herbal medicinal product usage among patients from Unified Health System is low and mainly affects women, individuals with mental diseases, and non-white people. Despite prescriptions by healthcare professionals, self-medication remains prevalent and increases the risk of drug interactions and therapeutic redundancy. Herbal medicinal products options within the Unified Health System are limited, mostly obtained from private pharmacies, and are linked to patients with a high economic status.

## Highlights

- Herbal medicinal product use was 3.8%, with high formal prescription; however, it is accompanied by a high risk of interactions and redundancy.
- Unified Health System pharmacies are not the main source of herbal medicinal products, suggesting low service implementation.
- Women, people with mental disorders, and allopathy simultaneously users should receive attention.
- Non-white people are less likely to use herbal medicinal product, suggesting inequalities in accessing these treatments.

## Graphical abstract

**Figure:**
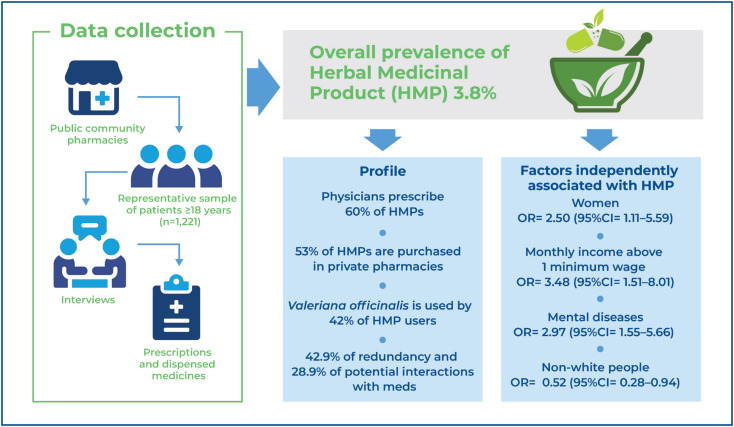

## INTRODUCTION

A medicinal plant is defined as a "species of the plant kingdom, whose parts (flowers, leaves, roots, stems, fruits, or seeds) are directly used or indirectly used in a preparation such as a medicine to treat a condition or disease".^(1)^ Meanwhile, herbal medicinal products (HMPs) are "any medicinal products, exclusively containing as one or more herbal substances or one or more herbal preparations, or one or more such herbal substances in combination with one or more such herbal preparations as active substances".^(2)^

Brazil has a rich tapestry of influence from indigenous and colonizing cultures on traditional medicine. Despite this historical background, traditional medicinal practices have declined with the advent of modern medicine, presenting persistent challenges in promoting and encouraging their appreciation and usage.^(3)^ As a strategy initiative, Brazil is among the nations that have formulated guidelines for the incorporation of medicinal plants into the public system, to enhance the safety, quality, and efficacy of traditional and complementary medicine (TM&C), aligning with recommendations from the World Health Organization.^(4)^ These guidelines aim to integrate TM&C into national health systems through established policies. Brazil's regulatory framework was delineated by the National Policy on Integrative and Complementary Practices (PNPIC - *Política Nacional de Práticas Integrativas e Complementares*, Ordinance nº 971, dated May 3, 2006).^(4)^ This policy legitimizes services and practices, including phytotherapy, as integral components of the public services provided by the Unified Health System (SUS - *Sistema Único de Saúde*). Additionally, the National Policy on Medicinal Plants and Phytotherapy (PNPMF - *Política Nacional de Plantas Medicinais e Fitoterápicos*, Decree No. 5,813, of June 22, 2006) strives to ensure safe access and the rational use of medicinal plants.^(5)^

Notwithstanding the comprehensive guidelines outlined in the PNPIC, which allocate responsibilities to federal, state, and municipal entities, aim to diversify therapeutic options for SUS users and facilitate access to HMPs. Only a limited number of municipalities have standardized, regulated,^(6)^ and implemented such programs within their healthcare facilities.^(7)^

A cross-sectional National Health Survey conducted in 2013 among Brazilian adults revealed that the prevalence of HMP use over the previous 12 months was 2.5%. However, this survey did not specifically identify factors associated with the use of phytotherapy.^(8)^ Generally, prevalence studies concerning HMP use in Brazil have focused on specific subpopulations, such as the elderly, children, and women,^(9,10)^ or have focused on particular diseases.^(11,12)^

The increasing market value of HMPs in Brazil underscores the growing acceptance of natural products for disease treatment. However, there remains a need for comprehensive information regarding HMP consumption and its associated factors in Brazil^(13)^ even nearly 17 years after the implementation of policies aimed at promoting its use within the SUS.

## OBJECTIVE

Our novel study aimed to determine the prevalence and associated factors of herbal medicinal product use among primary healthcare patients in southeastern Brazil.

## METHODS

### Research context and study area

Primary healthcare (PHC) in Brazil is provided through PHC units and family health programs.^(14)^ Primary healthcare units offer essential healthcare services to Brazilian citizens, including medical consultations, vaccinations, and the provision of medicines for several chronic conditions such as hypertension, diabetes, and rhinitis, which are provided free of charge to the population through public community pharmacies.^(15)^ Typically, public community pharmacies include a designated area for dispensing medicines, situated within the healthcare center itself or in a separate location. It is staffed by pharmacists and other professionals such as nurses.^(16)^

The data for this study were obtained from the Prover Project, an exit survey conducted in the context of Primary Healthcare in a southeastern Brazilian city located in the state of Minas Gerais.^(17)^ The city has a population exceeding 200,000 and holds the tenth position among the most populous cities in the state of Minas Gerais.^(18,19)^ With a Human Development Index of 0.764, it was recognized as a healthcare pole reference for other municipalities in the region.^(19)^ The city is well equipped with a healthcare infrastructure and has a substantial pool of human resources available for healthcare delivery at all levels of the SUS.^(20)^ It has five public community pharmacies located in its health districts that are responsible for providing prescription medicines; all have a pharmacist.^(20-22)^

### Data collection and recruitment

The Prover Project also aimed to evaluate the provision and use of medicines, prescription, and dispensing practices, and the level of knowledge of patients about their pharmacotherapy, and to determine the prevalence and associated factors of out-of-pocket pharmaceutical expenditure among primary healthcare patients.^(20-22)^ The data for this study were collected between August and November 2017, employing a multidimensional instrument that had undergone pretesting and validation and was developed by the research team based on relevant literature sources.^(23-26)^ The questionnaire was structured into the following domains: (1) variables related to social capital; (2) variables related to health and lifestyle habits; (3) variables related to expenses/costs of medications; (4) variables related to prescribed and non-prescribed medications as well as HMP; (5) variables related to medication storage; and (6) variables related to sociodemographic conditions.

This study primarily focused on HMP usage. This was evaluated through self-reported usage occurrences within the 15 days before the interview. Participants were asked the following question to ascertain this information: "In the last two weeks, did you use any herbal medicine? If YES: Can you tell me the name(s) of the herbal medicine(s) you used/are using in the last two weeks? (If you do not know, try to remember which plant it was made from).

The sample size was estimated considering Levin,^(27)^ equation (n=[z^2^α/2(pq)]/d^2^), resulting in a total of 1,067 individuals. This calculation considered a 50% prevalence of the events of interest, a 95% confidence level, and a 3% tolerated margin of error. A percentage of 15% was added to mitigate losses, resulting in a sample size of 1,228 individuals. Participants were then proportionally stratified into subpopulations based on the number of patients registered in each of the five public community pharmacies within the PHC system.^(20-22)^

The inclusion criteria for participants included a minimum age of 18 years, residence in the municipality, attendance at SUS primary health units for a minimum of six months, and the act of obtaining medication for themselves on the day of the interview.^(20-22)^ In addition to conducting interviews, the participants allowed us to copy data for each prescribed medication and information regarding the name of the medication, concentration, pharmaceutical form, and route of administration. Patients were excluded if they were not in the physical or mental condition to undergo the interview.

Individuals who declined to participate were invited to complete a condensed version of the questionnaire focusing on sex, age, and self-reported skin color. This allowed for a comparison of the profiles of the respondents and non-respondents.^(22)^

All dispensing service users were approached by interviewers and invited to participate after receiving their medication at public community pharmacies. Thus, 1,221 participants were included in this study.^(22)^

### Data preparation for analysis

The HMP names were verified through consultation with multiple guidelines and websites.^(28-31)^ Additionally, the medicinal indications cited by the survey respondents were standardized in scientific language.^(31,32)^

Our document analysis was based on a database of the medical prescriptions of HMP users. Subsequently, all medicinal plants based on their therapeutic indications^(30-32)^ and pharmaceutical medicines of HMP users were categorized using the Anatomical-Therapeutic-Chemical (ACT) coding system up to ATC level 1, as defined by the WHO Collaborating Center for Drugs Statistics Methodology.^(33)^

Additionally, we analyzed plant species that appeared in the National Relation of Medicinal Plants of Interest to the Unified Health System,^(34)^ National List of Essential Medicines (RENAME - *Relação Nacional de Medicamentos Essenciais*),^(35)^ and the Municipal List of Essential Medicines (REMUME - *Relação Municipal de Medicamentos Essenciais*).

### Data analysis

Indicators of redundancy and potential interactions were calculated to assess the quality of therapy by comparing HMPs with all pharmaceutical medicines (PMs) recorded in the prescriptions of HMP users.^(36-38)^

The proportion of redundancy was calculated using the following equation:^(36)^


$$\begin{array}{l} \text{Proportion of redundancy events}=\underline{\text{number of redundancy}}\\ \underline{\text{events/}}\text{total number of HMP users x 100/\%} \end{array}$$

To assess interactions between HMPs and pharmaceutical medicines used by patients, we relied on the "Lexi-Interact" platform,^(39)^ Stockley's book,^(32)^ and scientific literature. The potential interaction events were calculated using the following equation:^(36)^


$$\begin{array}{l} \text{Proportion of potential interaction events}=\underline{\text{number of}}\\ \underline{\text{interaction events/}}\text{total number of HMP users x 100/\%} \end{array}$$

### Associated factors with herbal medicinal products use dependent variable

The dependent variable was the use of herbal medicinal products in the past 15 days.

### Independent variables

The covariates included the following characteristics: sex (male, female), age (<60 and ≥60 years), marital status (not married/ married), self-reported skin color (white, non-white), educational level (0-3, 5-7, ≥8 years of study), monthly personal income (<1, 1 and ≥1 minimum wage), self-rated health status (excellent/ good and fair/ poor/ very poor), number of prescription drugs (1, 2-4, ≥5), all prescribed drugs obtained in SUS (yes, no), time required to arrive in to PHC unit (<15, ≥15 minutes of the travel time), self-reported hypertension (yes, no), self-reported anxiety, mood disorder or depression (yes, no) and self-reported diabetes (yes, no).

The diseases hypertension, anxiety, mood disorder, depression, and diabetes were identified through self-reporting to the following question: Has a physician ever stated that do you have or have had (disease)?

### Statistical analyses

Descriptive analysis was used to summarize the data related to the characteristics of HMP users, sources of recommendations and acquisitions, and the usage of each cited HMP. Bivariate and multivariate analyses were conducted, respectively, utilizing Pearson's χ^2^ test and logistic regression to assess the associations between variables and the usage of HMPs. To determine variables with significance in bivariate analyses, p≤0.2 from the Pearson's χ^2^ test was employed as a threshold. These were subsequently tested in the multivariate model. The best multivariate model was chosen through backward elimination, and p<0.05 was adopted as a criterion for the permanence of the factor in the adjusted models. Following the fitting of the logistic regression model, a global goodness-of-fit test was conducted to assess overall model performance. The results are presented as odds ratios (OR) with corresponding 95% confidence intervals (95%CI). All statistical analyses were performed using Stata version 15.1 (Stata Corporation, College Station, TX, USA).

### Ethics approval

Ethical approval was obtained from the Ethics Research Committee of *Fundação Oswaldo Cruz*, Brazil (CAAE: 49230115.6.0000.5091; #1.395.369. Participants were recruited voluntarily and provided written informed consent before the data collection commenced.

## RESULTS

### Prevalence of herbal medicinal products use and demographic characteristics

A total of 1,221 participants participated in the study (Table 1), and 47 reported the use of at least one HMP in the last 15 days, resulting in a prevalence of 3.8%. Most participants were women (65.2%), non-white (53.3%), older than 60 years (55.0%), married (57.6%), and earned up to one minimum wage (65.4%). More than half of the respondents did not have health plans, health insurance coverage (71.7%), or hypertension (71.3%).

**Table 1:** Prevalence of herbal medicinal products use and characteristics of respondents

Characteristic	n=1,221 n (%)
Herbal medicinal products use (Yes)	47 (3.8)
Sex (Female)	796 (65.2)
Age (≥60)	670 (55.0)
Marital status (With marital relationship/ married)	701 (57.6)
Self- reported skin color/ Race (Non-white)	625 (53.3)
Educational level (4 to 7 years of study)	483 (39.9)
Monthly personal income (Up to 1 minimum wage)*	457 (65.4)
Self-rated health status (Fair/ poor/ very poor)	691 (57.4)
Health plan or health insurance coverage (No)	876 (71.7)
Number of prescription drugs in the last 15 days (≥5)	522 (42.8)
All prescribed drugs obtained in SUS (No)	858 (70.4)
Time required to arrive in PHC unit (≥15 min)	759 (62.4)
Hypertension (Yes)†	870 (71.3)
Anxiety, mood disorder or depression (Yes)†	518 (42.4)
Diabetes (Yes)†	374 (30.6)

* In times the monthly Brazilian minimum wage (total of approximately 236.00 EUR) during the study period

† Condition diagnosed by a physician and self-reported by the patient in the interview.

SUS: *Sistema Único de Saúde*; PHC: primary healthcare.

### Herbal medicinal products use among primary health care patients

We identified 14 different HMPs consumed by patients with PHCs (Table 2). *Valeriana officinalis* (n=22, 42.3%) and *Ginkgo biloba* (n=8, 17.0%) were the most commonly used HMPs.

**Table 2:** Popular and Latin names of cited herbal medicinal products by users. Relative frequencies, self-reported indications, and the potential redundancies and interaction events

Popular name	Latin name	Frequency of use n (%)	Self-reported indications
Valerian* †	*Valeriana officinalis* L.	22 (42.3)	Sedative-hypnotic to treat stress and insomnia, and as an anxiolytic agent
Ginkgo* †	*Ginkgo biloba* L.	8 (17.0)	To improve cognitive function to treat memory, dementia, and cognitive disorders
Horse chestnut†	*Aesculus hippocastanum* L.	3 (6.4)	To treat venous insufficiency and capillary fragility (varicose veins, venous ulcers, hemorrhoids) and inflammation
Leather hat*	*Echinodorus macrophyllus* (Kunth) Micheli	3 (6.4)	To treat inflammation and as diuretic (edema) agent
"Guaco" and combinations*	*Mikania glomerata* Spreng	3 (6.4)	To treat respiratory disorders with productive cough. Bronchodilator and expectorant agent
Thorn-saint*	*Maytenus ilicifolia* Mart. ex Reissek	2 (4.2)	Anti-dyspeptic, antacid and gastric mucosa protector agent
Peppermint	*Mentha x piperita* L.	2 (4.2)	Antispasmodic and antiflatulence agent
Passiflora and combinations* †	*Passiflora incarnata* L.	2 (4.2)	To treat anxiety and insomnia and as a sedative agent
Mountain arnica	*Arnica montana* L.	1 (2.1)	Anti-inflammatory to treat injuries such as bruises, distortions, and sprains
Turmeric	*Curcuma longa* L.	1 (2.1)	Anti-dyspeptic, anti-inflammatory, liver protecting agent, and chemopreventive effects for cancer
Devil's claw	*Harpagophytum procumbens* DC. ex Meisn	1 (2.1)	To relieve moderate pain
Black mulberry	*Morus nigra* L.	1 (2.1)	Hormonal replacement, diuretics and anti-inflammatory
White sucupira	*Pterodon emarginatus* Vogel	1 (2.1)	To treat inflammation in bursitis, arthritis, rheumatism, healing of wounds and burns
Pomegranate	*Punica granatum* L.	1 (2.1)	To treat inflammatory disorders and as an antiseptic of the oral cavity
Total	NA	51 (100.0)	NA

* Redundancy event (yes); total n=6;

† Interaction event; total n=4.

NA: not applicable.

In this study, the participants reported using *V. officinalis* L. for various purposes, including treating anxiety, sleep disorders, and depression, promoting calmness, and alleviating palpitations. Additionally, *G. biloba* L. has been used to address conditions such as labyrinthitis, cramps, and pain, as well as to enhance memory and improve blood circulation (Table 2).

### Redundancy and potential interaction events

The occurrences of redundancy and potential interaction events between pharmaceutical medicines (PMs) concurrently prescribed with HMPs were 42.9% and 28.6%, respectively (Table 2). Based on scientific literature, we identified redundant drug pairs between the prescribed HMPs and PMs such as *Echinodorus macrophyllus* with furosemide, *Maytenus ilicifolia* with omeprazole, *Passiflora incarnate* with *clonazepam*, and *V. officinalis* with clonazepam.

Furthermore, we identified the potential clinical risks associated with certain combinations. These included an increased risk of bleeding when *A. hippocastanum* or *G. biloba* were used in conjunction with acetylsalicylic acid. This type of potential risk has also been identified for *G. biloba*, clopidogrel, and dabigatran.

Additionally, there was a risk of heightened sedative effects or strong drowsiness and dizziness when *P. incarnata* or *V. officinalis* was combined with clonazepam (Table 3).

**Table 3:** Potential clinical risks associated with redundancy and interaction events between herbal medicinal products and prescribed pharmaceutical medicines

Plant species	Prescribed pharmaceutical medicine	Potential clinical risks
*Aesculus hippocastanum L.*	Acetylsalicylic acid	Increases bleeding risk
*Ginkgo biloba L.*	Acetylsalicylic acid	Increases bleeding risk
	Clopidogrel	
	Dabigatran	
*Passiflora incarnata* L. and combinations	Clonazepam	Increases of sedative effect
*Valeriana officinalis* L.	Clonazepam	Increases strong drowsiness and dizziness

### Source of recommendations and acquisition of herbal medicinal products

**Figure 1:**
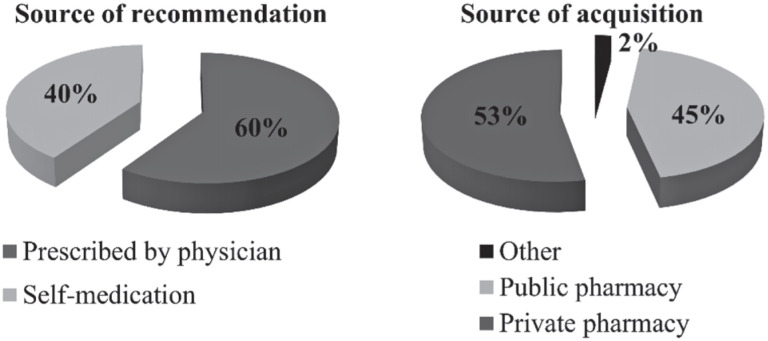
Source of recommendations and acquisition of herbal medicinal products

As depicted in figure 1, most HMPs were prescribed by physicians (60%) and used for self-medication (40%). Private pharmacies served as the primary source of HMPs (53%), followed closely by SUS public community pharmacies (45%) and other categories (2%).

### Associated factors with HMP use

Table 4 summarizes the factors associated with HMP use. Based on our results of the multivariate logistic regression model (Table 4), being a female participant (OR= 2.50; 95%CI= 1.11-5.59), having a monthly personal income above 1 minimum wage (OR= 3.48; 95%CI= 1.51-8.01), or having anxiety, mood disorder or depression (OR= 2.97; 95%CI= 1.55-5.66,) were positively associated with HMP use (p<0.05) whereas being non-white (OR= 0.52; 95%CI= 0.28-0.94) was negatively associated with HMP use (p<0.05).

**Table 4:** Results of the bivariate and multivariate analyses from the herbal medicinal products users (n=1,221)

Variable		HMP users	Bivariate analysis	Multivariate analysis
		Total n (%)	OR (95%CI)	OR (95%CI)
Sex				
	Male	8 (17.0)	Ref.	Ref.
	Female	39 (83.0)	2.60 (1.21-5.57)	2.50 (1.11-5.59)
Age (Years)				
	<60	19 (40.4)	Ref.	
	≥60	28 (59.6)	0.69 (0.36-1.31)	
Marital status				
	Not married	26 (55.3)	Ref.	
	Married	21 (44.7)	0.59 (0.33-1.05)	
Self-reported skin color				
	White	30 (63.8)	Ref.	Ref.
	Non-white	17 (36.2)	0.50 (0.27-0.90)	0.52 (0.28-0.94)
Educational level (years of study)				
	0-3	10 (21.3)	Ref.	
	5-7	19 (40.4)	1.05 (0.49-2.27)	
	≥ 8	18 (38.3)	1.04 (0.48-2.26)	
Monthly personal income*				
	<1	8 (17.0)	Ref.	Ref.
	1	18 (38.3)	1.65 (0.72-3.80)	2.03 (0.88-4.68)
	>1	21 (44.7)	2.10 (0.93-4.74)	3.48 (1.51-8.01)
Self-rated health status				
	Excellent/good	22 (46.5)	Ref.	
	Fair/poor/very poor	25 (53.5)	0.85 (0.47-1.55)	
Number of prescription drugs in the last 15 days				
	1	8 (17.0)	Ref.	
	2-4	19 (40.4)	0.96 (0.42-2.20)	
	≥ 5	20 (42.6)	1.36 (0.60-3.08)	
All prescribed drugs obtained in SUS?				
	No	34 (72.3)	Ref.	
	Yes	13 (27.7)	0.91 (0.48-1.72)	
Time required to arrive in to PHC unit				
	<15 minutes of the travel time	21 (44.7)	Ref.	
	≥15 minutes of the travel time	26 (55.3)	0.75 (0.42-1.33)	
Hypertension†				
	No	12 (25.5)	Ref.	
	Yes	35 (74.5)	1.18 (0.61-2.27)	
Anxiety, mood disorder or depression†				
	No	14 (29.8)	Ref.	Ref.
	Yes	33 (70.2)	3.20 (1.71-5.98)	2.97 (1.55-5.66)
Diabetes†				
	No	31 (66.0)	Ref.	
	Yes	16 (34.0)	1.17 (0.64-2.14)	

* In times the monthly Brazilian minimum wage (total of approximately 236.00 EUR during the study;

† Condition diagnosed by a physician and self-reported by the patient in the interview.

Ref: Reference category; OR: odds ratio; 95%CI: 95% confidence interval; PHC: primary healthcare; HMP: herbal medicinal products.

## DISCUSSION

The results of our study were obtained from a representative sample of adults aged 18 and above who used PHC units in the SUS from a southeastern Brazilian city. We observed a prevalence of 3.8% of HMP use, consistent with the findings of 2.5% for Brazil and 2.6% for the Southeast Region reported in the National Health Survey 2013, which was conducted with 145,580 Brazilians aged over 18 years living in a private household.^(8)^ The prevalence of HMP use by patients in PHC units in Brazil is high and may vary widely. However, studies conducted in small regions often do not investigate the associated factors or specify the timing of the questions used to assess the prevalence of HMP.^(40-42)^

Our study was conducted in one of the 564 municipalities in Minas Gerais, which offers Integrative and Complementary Practices for SUS patients.^(43)^ This particular municipality is a health pole for others in the same region and the majority of participants reported that HMPs were recommended by healthcare professionals through SUS facilities, indicating that few HMPs used were not available in public community pharmacies. We observed that two-fifths of the HMPs were not recommended by a qualified professional but rather chosen based on the empirical knowledge of friends, neighbors, or relatives, or used at individual discretion. This suggests that implementation is in its early stages, with high self-medication rates and extremely limited availability of HMPs, which are primarily obtained from private pharmacies.

Among the 14 HMPs reported by participants (Table 1), four - *Valeriana officinalis, Ginkgo biloba*, *Aesculus hipposcastanum*, and *Echinodorus macrophyllus* - collectively accounting for nearly 70% of the mentions were absent from the RENISUS list.^(34)^ *E. macrophyllus, Mikania glomerata*, *Maytenus ilicifolia, Mentha piperita, and Harpagophytum procumbens* were not found in Memento Fitoterápico.^(31)^ However, only these four HMPs are listed in the National List of Essential Medicines^(35)^ and only *V. officinalis* has been incorporated into the REMUME of this particular southeastern Brazilian city. These guidelines or listings support the development of herbal medicines available for public use, as well as to guide prescriptions and ensure safe and qualified access to medication for the population. *V. officinalis* and *G. biloba*, the most frequently reported HMPs, have self-reported indications aligned with national guidelines^(30,31)^ and are among the plant species with the highest number of registered phytotherapies in Brazil. *G. biloba* is one of the top 10 best-selling phytotherapies in 2023.^(44)^

We identified redundant and potential interactions between concurrently prescribed PMs and HMPs at rates of 42.9% and 28.6%, respectively. The term drug-drug interaction is employed when a patient is administered two or more drugs simultaneously, and the effects of these drugs may be intensified or weakened, potentially leading to side effects.^(45)^ The concept of redundancy effect arises when a patient uses multiple medications within the same therapeutic class prescribed for the same clinical condition, as per the Anatomical-Therapeutical-Chemical classification system.^(46)^ Both redundancy and interaction scenarios can pose potential clinical risks, serving as indicators of consumption and reflecting prescription quality.^(37,38)^ In Brazil, there are a limited number of studies on drug interactions within PHC^(47,48)^ however, a recent study conducted within the population residing in the catchment area of a rural Family Health Care Unit in a southern Brazilian county revealed approximately 36% potential drug interactions, which corroborates our data.^(49)^

Our multivariate analysis revealed that HMP usage is associated with the female sex and a personal monthly income above one minimum wage. Predictive characteristics also included a non-white ethnicity and having anxiety, mood disorders, or depression. Direct comparisons with previous studies conducted in Brazil are limited because of the methodological differences. Additionally, adults with anxiety, mood disorders, or depression were significantly more likely to use HMPs, which is consistent with a high prevalence of these disorders in Brazil, particularly among women.^(50)^ However, studies on the predictors of HMP usage in individuals with depression and mood disorders are limited.

The study has a few limitations. This study employed a cross-sectional design that is subject to bias. However, we mitigated these biases by recruiting, training, and supervising the field team to ensure compliance with the study protocol, conducting preliminary tests and pilot studies of the instruments, and closely monitoring the data collection process.^(21)^ Our enrollment strategy was proportional to users across all PHC units in the municipality, and we adopted standardized operational procedures for participant enrollment to avoid selection bias. Information bias was minimized by including participants who had been users of public community pharmacies for at least 6 months and immediately post attendance in pharmaceutical services.^(21)^ Additionally, we provided a clear definition of HMPs and collected consumption data over the last 15 days to reduce memory bias. Our findings are broadly applicable, especially to Brazilian municipalities with populations ranging from 100,000-900,000 inhabitants, as our sample was representative and reflected the adult population served in Brazilian PHC,^(51)^ with insignificant differences between participant and non-participant profiles.

## CONCLUSION

Our findings revealed that even in a health-referring municipality in southeastern Brazil, herbal medicinal product usage is low. Furthermore, the municipal SUS offers few options for herbal medicinal products. This suggests that prescriptions by health professionals and knowledge of this therapy by patients are still limited. Despite low overall usage, many patients used herbal medicinal products without supervision, which could expose them to redundancy and interaction events. Hence, it is crucial to underscore the importance of monitoring patients exposed to both herbal medicinal products and allopathic medications, as there may be potential clinical risks. Additionally, access to this type of treatment is restricted, primarily benefiting individuals with a higher socioeconomic status and women with self-reported anxiety, mood disorders, or depression may receive it as adjuvant therapy. This further underscores the need to invest in local policies to improve the supply, prescriptions, and use of herbal medicines. Based on our data, this study serves as a model for other locations with similar scenarios to reflect on their practices and take advantage of this diagnosis for improvement.
